# Associations Between Sugars Intakes and Urinary Sugars Excretion and Carbon Stable Isotope Ratios in Red Blood Cells as Biomarkers of Sugars Intake in a Predominantly Māori Population

**DOI:** 10.3389/fnut.2021.637267

**Published:** 2021-06-30

**Authors:** Lisa Te Morenga, Devonia Kruimer, Rachael McLean, Amandine J. M. Sabadel, Robert van Hale, Xavier Tatin, Jennié Harre Hindmarsh, Jim Mann, Tony Merriman

**Affiliations:** ^1^Department of Human Nutrition, University of Otago, Dunedin, New Zealand; ^2^Riddet Centre of Research Excellence, University of Otago, Dunedin, New Zealand; ^3^Edgar Diabetes and Obesity Research, University of Otago, Dunedin, New Zealand; ^4^Department of Preventive and Social Medicine, University of Otago, Dunedin, New Zealand; ^5^Department of Chemistry, University of Otago, Dunedin, New Zealand; ^6^AgroParisTech, Paris, France; ^7^Ngāti Porou Hauora Charitable Trust, Te Puia Springs, New Zealand; ^8^Department of Medicine, University of Otago, Dunedin, New Zealand; ^9^Department of Biochemistry, University of Otago, Dunedin, New Zealand

**Keywords:** added sugars, free sugars, carbon stable isotope ratio, urinary sugars, urinary excretion, Māori, New Zealand, dietary biomarker

## Abstract

Determining the extent to which added sugars intake contribute to non-communicable disease in various populations is challenging because it is difficult to accurately measure intakes. Biomarkers may provide a reliable and easily measured method of assessing intakes. In a predominantly Māori population we compared various sugars intake estimates derived from a 36 item sugar-specific food frequency questionnaire (FFQ) with biomarkers of sugars intake; urinary sugars excretion in random spot collections (*n* = 153) and carbon stable isotope ratios (*n* = 36) in red blood cells (RBCs, δ^13^C_RBC_) and in the alanine fraction of the RBCs (δ^13^C_alanine_). Estimated 24 h urinary sucrose+fructose excretion was statistically significantly correlated with intakes of total sugars (*r* = 0.23), sucrose (*r* = 0.26) and added sugars from sugar-sweetened beverages (SSBs; *r* = 0.26). δ^13^C_alanine_ was correlated with added sugars (*r* = 0.40). In log linear multiple regression models adjusted with HbA1C and eGFR δ^13^C_alanine_ predicted added sugars intakes (*r*^2^ = 0.29) and estimated 24 h urinary sucrose+fructose excretion predicted intakes of total sugars (*r*^2^ = 0.14), sucrose (*r*^2^ = 0.17), added sugars (*r*^2^ = 0.17) and sugars from SSBs (*r*^2^ = 0.14). These biomarkers have potential for improving assessment of sugars intake in New Zealand populations enabling monitoring of the effectiveness of sugar reduction strategies designed to reduce risk of NCDs. However, further validation is required to confirm these preliminary findings.

## Introduction

Sugars added to the diet are often referred to as added sugars or free sugars. Added sugars are defined as “all monosaccharides and disaccharides added by manufacturer, cook or consumer to sweeten foods or drinks including, sucrose, glucose, honey, syrups, but excludes fruit juices and fruit concentrates. Free sugars include added sugars plus fruit juices and fruit juice concentrates.” (1). There is widespread consensus that intakes of added or free sugars should be limited to <10% of total energy intake (1–3) based on evidence that high intakes contribute to excess weight gain (4) and dental caries (5), and are associated with increased risk of non-communicable diseases including type 2 diabetes (6) and cardiovascular disease (CVD) (7, 8). Māori are disproportionally affected by obesity, diabetes and CVD. While socioeconomic factors are major determinants (9), it is likely that excess consumption of added sugars in sugar-sweetened drinks and processed foods contribute to this disease burden. However, determining the extent to which added sugars contribute to disease in various populations is challenging because it is difficult to accurately measure intakes. Dietary assessment at a population level is still largely dependent on self-report methods such as 24 h diet recalls or food frequency questionnaires which are subject to reporting biases (10). Previous research has shown that self-reported intakes of sugars are particularly prone to misreporting (11). Biomarkers of dietary sugars intake may provide an alternative and more reliable method of assessing intakes. This will improve our understanding of the contribution of added sugars intakes to non-communicable diseases in different population groups and our ability to monitor the effectiveness of strategies to reduce added sugars intakes.

Two promising biomarkers of sugars intake have been identified, and validation studies of these biomarkers have developed regression equations to enable these measures to be converted into estimates of sugars intakes (12, 13). The first predictive biomarker assessed sugars excreted in 24-h urine samples. Results from controlled-feeding studies showed that 24-h urinary excretion of fructose and sucrose provided a valid method of measuring intake of total sugars in controlled-feeding studies (13–15), findings supported in cross-sectional studies (16). Applied in the Norfolk cohort of the European Prospective Investigation into Cancer and Nutrition (EPIC), sugars concentrations in spot urine collections were associated with the development of obesity (17, 18). Twenty-four hour urinary fructose excretion has also been used to a limited extent in studies of children, showing moderate correlations between the biomarker and dietary sugars intake assessed by 3 day weighed food records (19).

The second biomarker of sugars intake are carbon stable isotope ratios (^13^C/^12^C ratio expressed as δ^13^C) measured in various tissues. The δ^13^C composition of foods reflects the isotope composition of the plant and animals it originates from (20) and the δ^13^C composition of corn- and cane-derived sugars is distinctive from other foods and sweeteners. Since humans are unable to change the δ^13^C composition of their tissues, the δ^13^C in various tissues may reflect the level of consumption of sweetened foods and drinks (21) In studies conducted in the USA δ^13^C values were shown to be moderately correlated with intakes of sweeteners derived from corn and cane in cross-sectional analyses in tissues including whole blood (22–24), serum (25–27), plasma (25, 28), red blood cells (RBCs) (12, 29–31) and hair (24, 29, 32). Precision may be further improved by two different approaches. The first involves measuring the δ^13^C in the alanine component of the target tissue (29). Such measurements are more complex and time consuming but have increased specificity for sugars intake because alanine is directly involved in sugars metabolism via the Cahill cycle, a shuttle of carbon between plasma glucose and alanine (33). Up to ~60% of alanine in blood tissues is estimated to originate from intake of dietary sugars. The second approach takes into account that dietary proteins from meat and fish are also an important source of ^13^C, and could confound the association between δ^13^C and sugars intake (31, 34). Nitrogen stable isotope ratios (^15^N/^14^N; expressed as δ^15^N) are increased with intakes of fish and meat but not with consumption of cane and corn-derived sugars. Therefore, a dual isotope model, using δ^13^C with inclusion of δ^15^N, has been proposed to control for these confounding dietary effects (12, 30, 31).

In the USA, sugars added to foods are commonly derived from sugar cane, corn and sugar beet (35) with only cane and corn sugars being isotopically distinct from all other plant-derived sweeteners. In contrast, the main sweetener used in foods and drinks commonly available in New Zealand is derived from sugar cane. Thus, δ^13^C is a promising biomarker for assessing sugars in New Zealand populations. To date no research has been published on the comparative performance of the urinary sugars excretion and δ^13^C as biomarkers for sugars intake in populations like New Zealand where cane sugars predominate.

The ongoing *Gout and Related Conditions in Tairāwhiti: Genes and Environment Study* is examining the genetic relationship between gout and other metabolic diseases such as type 2 diabetes and heart disease and the role environmental factors play in combination with the predisposing genetic factors. As part of this broader study we compared three biomarkers of sugars intake, urinary sugars excretion in random spot collections, carbon stable isotope ratios in red blood cells, and carbon stable isotope ratios in red blood cell alanine against sugars estimates derived from a culturally-appropriate validated semi-quantitative food frequency questionnaire (FFQ) in a predominantly Māori population.

## Materials and Methods

### Subjects

This cross-sectional study recruited a total of 175 participants aged 16 years and over, with and without gout, who were able to give written consent. Participants were recruited via the patients register of Ngāti Porou Hauora Charitable Trust (NPH), the Māori primary health organization (PHO) and health care provider for all in the Ngāti Porou rohe (tribal territory) on the largely rural East Coast area of the Tairāwhiti/Gisborne region in the North Island of New Zealand. Potential participants were either contacted by the research nurse via telephone or mail or approached in person at NPH health centres at Tawhiti, Ruatoria, Tokomaru Bay, Matakaoa, Uawa, and Puhi Kaiti (Gisborne). Further participants were recruited at community centres and through community groups, events and by word of mouth. The study protocol, risks and benefits were explained to each subject and written consent was given. The study was approved by the University of Otago Human Ethics Committee (13/117). The study was overseen by the NPH Research Coordinator Dr. Harré Hindmarsh, and the research team was advised by the NPH Gout and Related Conditions Research Advisory Group, chaired by Research Coordinator and consisting of a NPH general practitioner, nurse, manager, two community representatives, Professor Merriman, and the research nurse (study recruiter).

Data collection took place between November 2013 and March 2015. To reduce attrition rates, data were collected at a time and location convenient to the participant, usually during daytime working hours and either at local health clinics or in the participants' homes.

### Experimental Protocol

Participants completed a sugars-specific FFQ in the presence of the research nurse who was able to provide clarification of questions if necessary. Two 10 ml urine specimen collection containers were provided to the participants for a spot urine collection at the clinic appointment. One container was for analyses of urinary creatinine and urate. The other for urinary sugars analyses and contained 30 mg boric acid as a preservative. Four blood samples were collected in two serum separator vacutainers, for serum analysis, and two vacutainers containing EDTA (an anticoagulant for blood samples) for analysis of plasma and RBCs. A general questionnaire was administered by the research nurse, registered and trained in rural health care, to elicit information on variables including age; sex; educational attainment; employment status; smoking habits; previous diagnosis of metabolic disorders; medical therapies including uric acid-lowering medication, cholesterol lowering medications, diuretics and other antihypertensive medication; family history of gout and diabetes; alcohol and seafood consumption; and physical activity level. Height (m), weight (kg), and waist circumference (cm) were also measured by the research nurse. Missing data were obtained from patient medical records with participant permission.

### Assessment of Dietary Intakes

Participants completed a 34-item, semi-quantitative FFQ to assess sugars intake over the past month, which was developed and validated previously in this Māori community (36). Usual daily intakes of total available sugars; added sugars; added sugars in sugar-sweetened beverages; and sugars from fruits were calculated via a pre-developed spreadsheet estimated using Kaiculator^©^ 2013 analysis software and New Zealand food composition data [2010 NZ FOODfiles; (37)].

The FFQ was validated is a previous study conducted by Masters of Dietetics students. Cross-classification agreement of sugars intake quartiles from FFQ and repeated 24-h recalls in 72 participants showed that 95–97% of participants were classified into the same or adjacent quartiles, with weighted kappa values (K_w_) ranging from 0.43 to 0.51, which suggests a moderate agreement between the two dietary assessment techniques (36). Correlation coefficients between the FFQ and repeated 24-h recalls ranged from 0.59 for total fructose intake, to 0.76 for total sugars from SSBs intakes.

Sugars intakes were defined six ways using data collected from the FFQ:

1. Total sugars: the sum of all sugars from all foods and beverages
2. Sucrose: the sum of sucrose from all foods and beverages.
3. Added sugars: the sum of all sugars minus lactose derived from beverages except 100% fruit juice, the sum of glucose, fructose and sucrose from dairy foods and total sugars in breakfast cereals, iceblocks, cakes, biscuits, confectionary and chocolate.
4. Added sugars from sugar-sweetened beverages (SSBs): the sum of total of fructose, glucose and sucrose from all beverages including fruit juices and alcoholic beverages.
5. Total sugars from sweetened foods (all food items in which sugars are added as a sweetener)
6. Totals sugars from all fresh raw fruit items.

### Laboratory Analyses

#### Blood collection

For serum analyses (lipids, urate, creatinine) blood samples were centrifuged in the field for 15 min at 3,000 rpm at 4°C. The samples were couriered to Dunedin and analysed by Southern Community Laboratories (SLC), an accredited diagnostic laboratory. Haemoglobin A_1C_ (HbA_1C_) was obtained from whole blood using ion-exchange HPLC (BioRad D-10™, Haemoglobin A_1C_ Program) at TLab, Gisborne. After analysis, the remaining blood sample was centrifuged and RBCs were washed twice with a saline solution (0.9 g sodium chloride (NaCl) with 100 mL deionised water) before transport to the Department of Human Nutrition at the University of Otago in Dunedin at 0°C and after arrival transferred to −20°C until analysis. Serum creatinine was measured by SCL using the 'Roche Cobas 8000 system. Estimated glomerular filtration rate (eGFR) was calculated using the Modification of Diet in Renal Disease formula (38).

#### Urinary Measurements

Urine samples were preserved by adding boric acid (30 mg). Urine samples were returned to the Department of Biochemistry at the University of Otago in Dunedin at room temperature. They were then temporarily stored (<2 days) at 4°C and transferred to long-term storage at −20°C in the Department of Human Nutrition laboratories until analysis. Spot urine collections samples were defrosted at room temperature for analysis. Urinary sucrose, fructose and glucose concentrations were estimated using spectrophotometry with an enzymatic kit (K-SUFRG, Megazyme International Ireland). The UV-method is based on the determination of D-glucose before and after hydrolysis of sucrose by β-fructosidase. D-fructose was determined after isomerization by phosphoglucose isomerase. The smallest differentiating absorbance for the assay is 0.010 absorbance units, corresponding to a concentration of 0.69 mg/L of glucose, fructose or sucrose. The initial protocol was modified to run on a microplate reader in 96-well plates. Each run included fructose, glucose and sucrose standards (50, 100, 200, 300, and 400 mg/L) and samples were measured in duplicates (*R*^2^ for the standard curve was 0.999 for fructose and 0.999 for sucrose for each of the plates measured). When the coefficient of variance (CV%) was more than 10%, samples were re-analysed. Urinary creatinine concentrations were measured using a Roche Modular P (Hitachi) analyser by SLC.

We estimated 24 h urinary excretion of the sum of sucrose and fructose from spot urine samples by creatinine adjustment with spot urinary creatinine concentration using the following formula:

$$\begin{array}{l} 24h\;urinary\;sucrose\;+\;fructose\;(mg)=spot\;urinary\;sucrose\\ +\;fructose\;(mg/L)/spot\;creatinine\;(g/L)\;X\;24h\;Creatinine\;(g) \end{array}$$

where 24 h creatinine was assumed to be 1.7 and 1.0 g for males and females, respectively.

#### Stable Isotope Measurements of Bulk RBCs

Stable isotope ratios were determined in a convenience sub-sample (the final 36 participants recruited for the study, and from whom we had RBCs) to pilot test the suitability of the method for assessing sugars intakes in New Zealand population. Bulk carbon (δ^13^C_RBC_) and nitrogen (δ^15^N_RBC_ – measured to adjust for potential confounding by meat and fish intakes) isotopic compositions were determined on ~0.8 mg of freeze-dried RBCs, weighed into tin capsules. δ^13^C_RBC_ and δ^15^N_RBC_ were determined by combustion in a NA 1500 Elemental Analyser (CE Instruments, Milan), and measurement of the resulting CO_2_ or N_2_ gases (respectively) by a Thermo Finnigan Delta Advantage Isotopic Ratio Mass Spectrometer (EA-IRMS) at the Isotrace lab facility (Dunedin, New Zealand). The conventional method of expressing δ^13^C or δ^15^N at natural abundance is in per mil (‰) abundance of ^13^C or ^15^N relative to an international standard (Vienna PeeDee Belemnite, VPDB or atmospheric N_2_, respectively), as follows:

(1) $$\begin{array}{l} \delta^{13}C=\;({(}^{13}C{/}^{12}C_{\text{sample}}{-}^{13}C{/}^{12}C_{\text{standard}}){/}^{13}C{/}^{12}C_{\text{standard}})\\ \;\times 1000‰\\ \delta^{15}N=\;({(}^{15}N{/}^{15}N_{\text{sample}}{-}^{15}N{/}^{15}N_{\text{standard}}){/}^{13}N{/}^{12}N_{\text{standard}})\\ \;\times 1000‰ \end{array}$$

The instrument precision was 0.2‰ for C and 0.2‰ for N, based on multiple measurements of laboratory control material (EDTA). Data were calibrated to the international scales using triplicate measurements of two reference materials (USGS41 and 41) run with each batch of samples.

#### Stable Isotope Measurements of Alanine in RBCs

Stable carbon isotope ratios of alanine in RBCs (δ^13^C_alanine_) were determined after extraction and derivatization of alanine using adapted protocols (39, 40). In brief, aliquots of 50 μL RBCs and 50 μL of internal standard were pipetted into a Kimax tube. Samples were hydrolysed with 1 mL 6 M HCl. The tube was then filled with N_2_, sealed (to prevent drying while heated), shaken, and heated at 150°C for 70 min. Samples were cooled down to room temperature, and centrifuged at 3,000 rpm for 7 min. The supernatant was transferred into a clean Kimax^©^ tube and evaporated to dryness at 60°C in a heating block under a gentle stream of N_2_. Alanine was then derivatised following the protocol by Styring et al. (41). δ^13^C_alanine_ was measured by gas-chromatography combustion isotope-ratio mass-spectrometer (GC-IRMS), using a Thermo Trace gas chromatograph, the GC-IsoLink combustion interface, and a Delta-XP isotope ratio mass spectrometer (Thermo Fisher Scientific). Two hundred nanoliters aliquots of derivitised alanine were injected at 270°C in splitless mode, carried by helium at 1.4 mL min^−1^ and separated on a VF-35 ms column (0.32 mm ID and a 1.0 μm film thickness). The oxidation reactor was set at 950°C and the reduction reactor was left at room temperature. Samples were analysed in duplicate along with amino acid laboratory standards of known isotopic composition (measured on EA-IRMS). Raw deltas or chromatographic peaks are measured against a CO_2_ monitoring gas and corrected to PDB with an internal standard of caffeine (δ^13^C = −26.98‰) (42). Derivatised δ^13^C_alanine_ was corrected relative to the δ^13^C_alanine_ of the laboratory standard to account for the exogenous C and kinetic fractionation introduced during derivatisation (42).

### Data Analyses and Statistics

All statistical analyses were performed using Stata/IC 14.2 for Mac (StataCorp, College Station, TX, USA). Descriptive data are presented as the mean and standard deviation (SD) unless specified otherwise. Sugars variables were log transformed to account for skewness in correlation and regression analyses. δ^13^C_RBC_ and δ^13^C_alanine_ variables were inversed (since the values are expressed as negatives) and then log transformed.

Partial correlation coefficients were calculated for the associations between the six definitions of sugars intakes (described above) as a continuous variable and the additive inverse of δ^13^C_RBC_ and additive inverse of δ^13^C_alanine_. Because the FFQ was designed to rank sugars intakes by quartiles rather than to provide validated estimates of actual intake 2 participants reported very high sugars intake values (e.g., >9 kg/d) therefore intakes were censored at 500 g/d. δ^15^N_RBC_ was included as the control variable to account for the effect of meat and fish intake on δ^13^C values.Partial correlation coefficients were also calculated for the associations between 24 h urinary sucrose + fructose and the additive inverses of δ^13^C_RBC_ and δ^13^C_alanine_ with δ^15^N_RBC_ included as the control variable. Finally correlation coefficients were calculated for the associations between 24 h urinary sucrose+fructose and sugars intakes.

Log linear multiple regression models were used to determine whether the three sugars intake biomarkers could predict the various measures of dietary sugars intakes. Single variable regression analyses were conducted to test for an association between sugars intake measures and each biomarker. Stepwise regression was used to identify whether covariates for δ^15^N_RBC_, age, sex, BMI, HbA1c and EGFR should be included in multivariate log regression models, up to a maximum of two covariates for the carbon stable isotope models and four covariates for the 24 h urinary sucrose + fructose model with *p* < 0.1. EGFR and HbA1C were selected to account for potentially abnormal urinary sugars excretion in people with impaired glycaemic control. We also tested the associations between the three biomarkers with regression analyses.

## Results

The study included 175 participants of whom 122 had a previous diagnosis of gout. Table 1 describes the participant characteristics. The mean (SD) age of the group was 60 (15) years and mean BMI was 35.2 (9.7) kg/m^2^ (Table 1). Seventy-three percent were obese (BMI >30 kg/m^2^). Eighteen percent of participants had hypertension stage 2 (systolic blood pressure ≥145 or diastolic blood pressure ≥90 mm Hg), 79% had high total cholesterol (>4 mmol/L), 32% had impaired glucose tolerance [HbA_1C_ between 40 and 50 mmol/mol; (43)] and 18% had HbA_1C_ above 50 mmol/mol, consistent with a diagnosis of diabetes (43), indicating a high level of comorbidities in the population. There were no statistically significant differences in variables between the complete sample population and the RBC subset (*n* = 36).

**Table 1:** Characteristics of study participants of the complete sample and the subset of participants with red blood cell samples.

	**Complete sample, *n* = 175**	**RBC subset, *n* = 36**
	**Mean (SD) or percent**	**Mean (SD) or percent**
Sex, *n*; % male	175 (67)	36 (72)
Age, y	60.4 (14.9)	60.7 (16.7)
Ethnicity (% Māori)	87%	89%
BMI, kg/m^2^	35.2 (9.2)	35.1 (8.5)
Waist circumference (cm)	112 (20)	112 (14)
Systolic blood pressure, mm Hg	135 (20)	138 (21)
Diastolic blood pressure, mm Hg	80 (14)	83 (12)
HbA_1C_, mmol/mol	45.9 (15.7)	45.7 (16.4)
Serum uric acid, mmol/L	0.43 (0.11)	0.43 (0.13)
Total cholesterol, mmol/L	5.00 (1.11)	4.80 (1.12)
Urinary creatinine, mmol/L	10.8 (5.9)	11.4 (8.0)
eGFR, mL/min/1.73 m^2^	69.5 (19.2)	65.6 (22.2)
Estimated 24 h urinary sucrose+fructose excretion (mg)^1^	4.7 (3.6, 4.1)^2^	6.5 (3.3, 12.7)^3^
δ^13^C_alanine_, ‰^4^	–	−19.68 (2.08)
δ^13^C_RBC_, ‰^5^	–	−22.64 (0.71)
δ^15^N_RBC_, ‰^6^	–	7.92 (0.47)

1 *Urinary excretion of sucrose and fructose measured in spot urine samples and adjusted to estimate 24 h excretion with spot urinary creatinine, reported as geometric mean (95% CI)*.

2 *n = 144*.

3 *Only n = 28 participants provided both urine and blood samples*.

4 *The ^13^C/^12^C ratio in red blood cell alanine*.

5 *The ^13^C/^12^C ratio in red blood cells*.

6 *The ^15N^/^14N^ ratio in red blood cells*.

**Figure 1:**
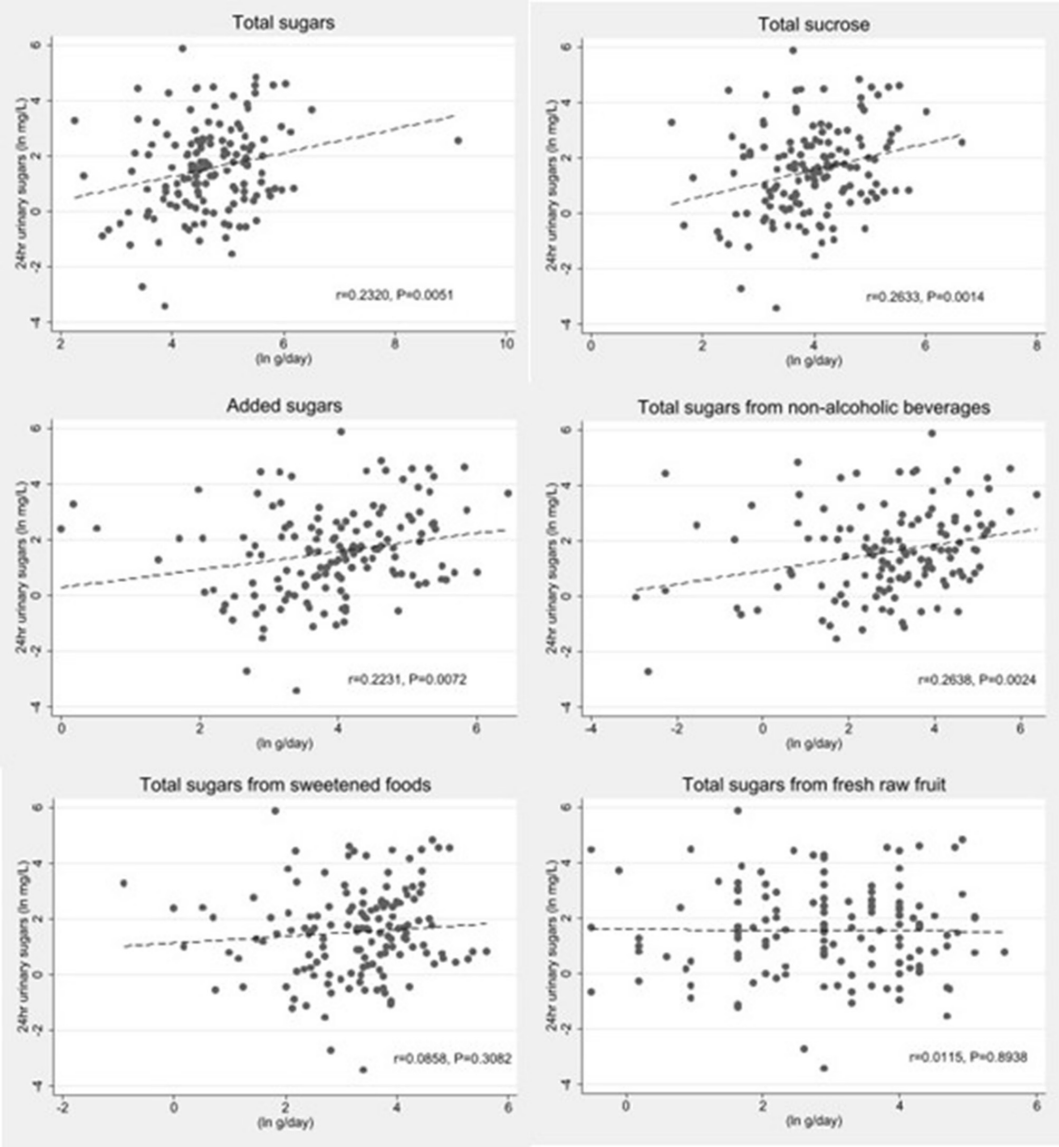
Scatter plots of log transformed estimated 24 h urinary sucrose+fructose excretion by log transformed sugars intakes estimated by a sugar specific FFQ (*n* = 140). The dashes lines represent the linear fit models.

Sugars intakes estimated by the food frequency questionnaire for six definitions for sugars are provided in Table 2. Extreme values reported by 2 individuals were truncated to a maximum value of 500g/day. Spot urine samples were available for 150 of the 175 participants recruited for the study and urinary creatinine was available for 144 of these. Excretion of estimated 24 h urinary sucrose + fructose was statistically significantly correlated with self-reported intakes of sucrose, added sugars, total sugars from SSBs and total sugars (*p* = 0.051) (Figure 1).

**Table 2:** Sugars intakes reported by six definitions estimated from a 36 item food frequency questionnaire (g/day).

	**Median**	**Min**	**Max**	**Interquartile range**
Total sugars^1^	99.6	9.5	500	(68.6, 184.4)
Sucrose^2^	55.9	1.0	500	(25.3, 101.8)
Added sugars^3^	54.2	4.3	500	(30.7, 85.9)
Added sugars from SSB^4^	20.3	0.0	500	(6.1, 63.3)
Total sugars from sweetened foods^5^	29.1	0.0	272.3	(12.3, 50.6)
Total sugars in raw fruit^6^	18.0	0.0	252.4	(5.2, 54.1)

*N = 175*.

1 *Total sugars content of all food items*.

2 *Total sucrose content of all food items*.

3 *Total sugars derived from beverages except 100% fruit juice minus lactose, plus the sum of glucose, fructose and sucrose content of dairy foods, and total sugars content in breakfast cereals, iceblocks, cakes, biscuits, confectionary and chocolate*.

4 *SSBs, sugar sweetened beverages; includes the total fructose, glucose and sucrose content in all beverages including fruit juices and alcoholic beverages*.

5 *Total sugars content in all sweetened food items*.

6 *Total sugars content in all fresh raw fruit items*.

**Figure 2:**
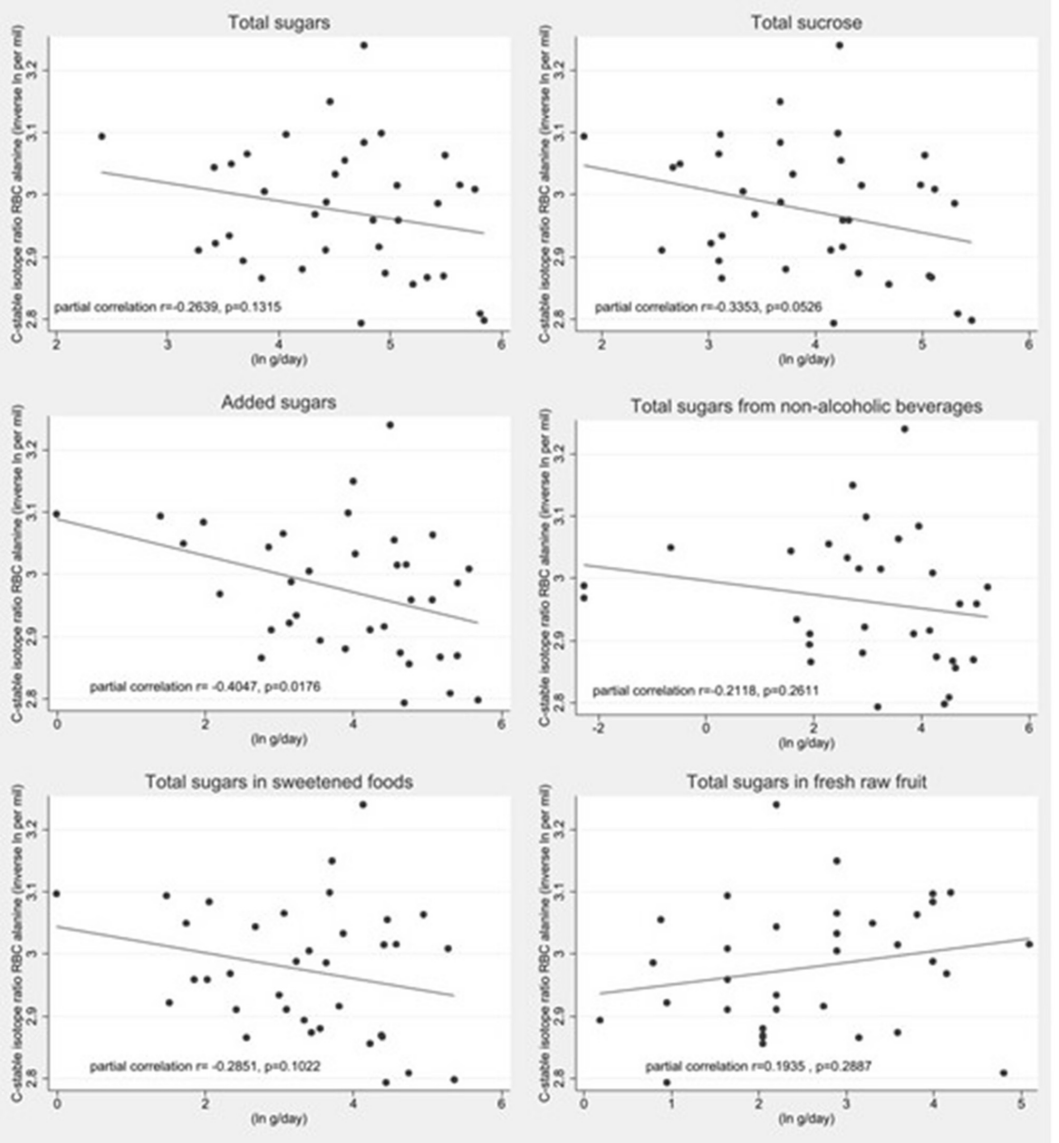
Scatter plots of the log transformed additive inverse δ^13^C_alanine_ values by log transformed sugars intake estimates estimated by a sugar specific FFQ (*n* = 36). The dashed represents the linear fit model. Partial correlation values after controlling for δ^15^N_RBC_ are presented within each plot.

We conducted isotope analyses on RBCs from a subsample of 36 participants. δ^13^C_RBC_ correlated with δ^15^N_RBC_ (*r* = 0.348, *p* = 0.038) but not with δ^13^C_alanine_ (*r* = 0.012, *p* = 0.948). δ^13^C_alanine_ correlated with intakes of total added sugars after controlling for δ^15^N_RBC_ but not for any other definitions of sugar intakes (Figure 2). δ^13^C_RBC_ was not correlated with any sugars intake variables. Estimated 24 h urinary sugars excretion correlated with δ^13^C_RBC_ (*r* = 0.41; *p* = 0.0385) but did not correlate with δ^13^C_alanine_ (*r* = 0.07; *p* = 0.7275) after controlling for δ^15^N_RBC_. Correlation coefficients between sugars intake variables and biomarkers are presented in Table 3.

**Table 3:** Correlation coefficients between dietary sugars intakes estimated by food frequency questionnaire (g/d) and biomarkers for sugars intake from spot urinesamples and red blood cells.

	**24 h urinary sucrose + fructose, mg^1^**		**δ^13^C_alanine_, ‰^2^**		**δ^13^C_RBC_, ‰^3^**		**δ^15^N_RBC_, ‰^4^**	
	***r***	***p***	***Partial r***	***p***	***Partial r***	***p***	***r***	***p***
Total sugars^5^	0.232	0.005	0.264	0.132	−0.087	0.620	−0.058	0.739
Sucrose^6^	0.263	0.001	0.335	0.053	−0.090	0.606	0.028	0.876
Added sugars^7^	0.223	0.007	0.405	0.018	−0.108	0.538	0.082	0.635
Added sugars from SSB^8^	0.264	0.002	0.212	0.261	−0.010	0.959	0.152	0.408
Total sugars from sweetened foods^9^	0.086	0.308	0.285	0.102	−0.303	0.077	−0.076	0.661
Total sugars in raw fruit^10^	−0.012	0.894	−0.194	0.289	0.009	0.963	−0.119	0.504

1 *Urinary excretion of the total of sucrose and fructose measured in spot urine samples and adjusted to estimate 24 h excretion with spot urinary creatinine; n = 144*.

2 *Partial correlation coefficient for the ^13^C/^12^C ratio in red blood cell alanine controlling for δ^15^N, n = 36*.

3 *Partial correlation coefficient for the ^13^C/^12^C ratio in red blood cells controlling for δ^15^N, n = 36*.

4 *Correlation coefficient for the ^15^N/^14^N ratio in red blood cells, n = 36*.

5 *Total sugars content of all food items*.

6 *Total sucrose content of all food items*.

7 *Total sugars derived from beverages except 100% fruit juice minus lactose, plus the sum of glucose, fructose and sucrose content of dairy foods, and total sugars content in breakfast cereals, iceblocks, cakes, biscuits, confectionary and chocolate*.

8 *SSBs, sugar sweetened beverages; includes the total fructose, glucose and sucrose content in all beverages including fruit juices and alcoholic beverages*.

9 *Total sugars content in all sweetened food items*.

10 *Total sugars content in all fresh raw fruit items*.

Unadjusted, single variable, log regression analyses showed a significant association between estimated 24 h urinary sucrose + fructose excretion and total sugars (*p* = 0.002), sucrose (*p* = 0.001), added sugars (*p* = 0.007) and sugars in SSBs (*p* = 0.003). There was also a statistically significant association between δ^13^C_alanine_ and added sugars (*P* = 0.025) and sucrose at the 90% confidence level (*P* = 0.08). There were no statistically significant associations between δ^13^C_RBC_ or δ^15^N_RBC_ and any sugars intake variables. There was a significant association between δ^13^C_RBC_ and 24 h urinary sucrose+fructose excretion (*p* = 0.049).

In multivariate log linear regression analyses including HbA1C and eGFR as covariates, estimated 24 h urinary sucrose + fructose excretion was a significant predictor of intakes of total sugars, sucrose, added sugars and sugars in SSBs (Table 4). The best predictive model was between 24 h urinary sucrose+fructose excretion and sucrose (*p* < 0.001) with the model explaining 17.4% of the variation in sucrose intake. For each 1% increase in 24 h urinary sucrose+fructose there was a 0.17% increase in sucrose intake (*P* < 0.0001).

**Table 4:** Associations between dietary sugars intakes (g/d) and estimated 24 h urinary sucrose+fructose excretion from a multivariate log linear regression model^1^.

	**24 h urinary sugar predictor value^2^**	***P* for 24 h urinary sugar**	**Obs**	**F(3, 26)**	***P* for model**	***R*^2^**	**Adjusted *R*^2^**
Total sugars^3^	0.14 (0.06, 0.22)	0.001	133	8.15	0.0001	0.1593	0.1398
Sucrose^4^	0.17 (0.08, 0.26)	<0.001	133	10.23	<0.0001	0.1922	0.1735
Added sugars^5^	0.18 (0.07, 0.29)	0.002	133	10.22	0.0001	0.1920	0.1732
Added sugars from SSB^6^	0.31 (0.11, 0.51)	0.003	133	7.31	0.0002	0.1589	0.1372
Total sugars from sweetened foods^7^	Not estimable						
Total sugars in raw fruit^8^	Not estimable						

1 *Models include covariates for HbA1C and estimated glomerular filtration rate (eGFR)*.

2 *Percentage change (95% CI) in independent sugars variable due to a 1% increase in δ ^13^C_alanine_ (^13^C/^12^C ratio in red blood cell alanine)*.

3 *Total sugars content of all food items*.

4 *Total sucrose content of all food items*.

5 *Total sugars derived from beverages except 100% fruit juice minus lactose, plus the sum of glucose, fructose and sucrose content of dairy foods, and total sugars content in breakfast cereals, iceblocks, cakes, biscuits, confectionary and chocolate*.

6 *SSBs, sugar sweetened beverages; includes the total fructose, glucose and sucrose content in all beverages including fruit juices and alcoholic beverages*.

7 *Total sugars content in all sweetened food items*.

8 *Total sugars content in all fresh raw fruit items*.

In multivariate log linear regression analyses including HbA1C and eGFR as covariates, δ^13^C_alanine_ was a significant predictor of added sugars intakes (Table 5). For each 1% increase in δ^13^C_alanine_ an increase in added sugars intake of 4.9% is predicted. The adjusted model explained 28.5% of the variation in added sugars intakes estimated by the FFQ also predicted total sugars intake from sweetened foods at the 90% confidence level (*p* = 0.072) in the HBA1c and eGFR adjusted model which was statistically significant (*p* = 0.0065) explaining 30% of the variation in sugars intake. δ^13^C_RBC_ did not predict sugars intakes in multivariate log regressions models (Table 6).

**Table 5:** Associations between dietary sugars intakes (g/d) and δ ^13^C_alanine_ from a multivariate log linear regression model^1^.

	**δ^13^C_alanine_ predictor value^2^**	***P* for δ^13^C_alanine_**	**Obs**	**F(3, 26)**	***P* for model**	***R*^2^**	**Adjusted *R*^2^**
Total sugars^3^	2.0 (−1.0, 5.0)	0.183	30	2.6	0.0733	0.231	0.1423
Sucrose^4^	2.7 (−0.4, 5.9)	0.09	30	3.19	0.0402	0.2691	0.1847
Added sugars^5^	4.8 (0.7, 8.9)	0.024	30	4.85	0.0082	0.3589	0.285
Added sugars from SSB^6^	4.1 (−3.7, 12.6)	0.296	30	0.99	0.4132	0.1148	−0.0007
Total sugars from sweetened foods^7^	3.6 (−0.3, 7.6)	0.072	30	5.12	0.0065	0.3713	0.2987
Total sugars in raw fruit^8^	−2.3 (−7.3, 2.8)	0.352	30	0.32	0.809	0.0373	−0.0783

1 *Models include covariates for HbA1C and estimated glomerular filtration rate (eGFR)*.

2 *Percentage change (95% CI) in independent sugars variable due to a 1% increase in δ^13^C_alanine_ (^13^C/^12^C ratio in red blood cell alanine)*.

3 *Total sugars content of all food items*.

4 *Total sucrose content of all food items*.

5 *Total sugars derived from beverages except 100% fruit juice minus lactose, plus the sum of glucose, fructose and sucrose content of dairy foods, and total sugars content in breakfast cereals, iceblocks, cakes, biscuits, confectionary and chocolate*.

6 *SSBs, sugar sweetened beverages; includes the total fructose, glucose and sucrose content in all beverages including fruit juices and alcoholic beverages*.

7 *Total sugars content in all sweetened food items*.

8 *Total sugars content in all fresh raw fruit items*.

**Table 6:** Associations between dietary sugars intakes (g/d) and δ ^13^C_RBC_ from a multivariate log linear regression model^1^.

	**δ ^**13**^C_**RBC**_ predictor value^2^**	***P* for δ^13^C_RBC_**	**Obs**	**F(3, 25)**	***P* for model**	***R*^2^**	**adjusted *R*^2^**
Total sugars^3^	−6.5 (−16.9, 5.1)	0.323	29	1.91	0.1541	0.231	0.1423
Sucrose^4^	−5.8 (17.2, 7.2)	0.438	29	1.81	0.1708	0.1786	0.1786
Added sugars^5^	−7.2 (−22.5, 11.1)	0.467	29	2.29	0.1027	0.2157	0.1216
Added sugars from SSB^6^	Not estimable						
Total sugars from sweetened foods^7^	−8.9 (−22.3, 6.7)	0.288	29	3.47	0.0311	0.2939	0.2092
Total sugars in raw fruit^8^	0.5 (−17.4, 22.3)	0.874	29	0.02	0.9966	0.0023	−0.1225

1 *Models include covariates for HbA1C and estimated glomerular filtration rate (eGFR)*.

2 *Percentage change (95% CI) in independent sugars variable due to a 1% increase in δ ^13^C_RBC_ (^13^C/^12^C ratio in red blood cells)*.

3 *Total sugars content of all food items*.

4 *Total sucrose content of all food items*.

5 *Total sugars derived from beverages except 100% fruit juice minus lactose, plus the sum of glucose, fructose and sucrose content of dairy foods, and total sugars content in breakfast cereals, iceblocks, cakes, biscuits, confectionary and chocolate*.

6 *SSBs, sugar sweetened beverages; includes the total fructose, glucose and sucrose content in all beverages including fruit juices and alcoholic beverages*.

7 *Total sugars content in all sweetened food items*.

8 *Total sugars content in all fresh raw fruit items*.

The predictive equation for added sugars intake based on δ^13^C_alanine_ is defined as follows:

$$\begin{array}{l} \text{Added sugars }(\text{g/d})=\text{exp}(-4.8042\times \text{ln}[\delta^{13}{\text{C}}_{\text{alanine}}\times -1]\\ +[0.0270777\times \text{eGFR}]-[0.0349989^{*}\text{HbA1C}]\;17.99757) \end{array}$$

Where δ^13^C_alanineis_ measured in mg, HbA1C in mmol/mol and eGFR in mL/min/1.73 m^2^

## Discussion

This is the first study to assess the association between self-reported sugars intake and two different biomarkers of sugars intake in a population of New Zealand Māori adults; urinary sucrose and fructose excretion, δ^13^C_alanine_ and δ^13^C_RBC_. We found that urinary sugars in spot urine samples were very weakly correlated with self-reported intakes of total sugars, sucrose, total added sugars and added sugars in SSBs. In the subset of the population in which we were able to conduct carbon stable isotope analyses we found that δ^13^C_alanine_ in RBCs was weakly correlated with self-reported intake of added sugars after partial adjustment for δ^15^N to account for potential confounding by meat and fish intake. In log linear multiple regression models adjusted with HbA1C and eGFR δ^13^C_alanine_ some predicted added sugars intakes and estimated 24 h urinary sucrose+fructose excretion predicted sucrose and added sugars intakes.

Several studies have shown 24 h urinary sucrose and fructose to be valid predictive biomarkers of sugars intake. In a 30 day controlled-feeding crossover study involving with 12 participants living in a metabolic unit and consuming three different diets varying in sugars content for 10 days each Tasevska et al. (13) showed a strong correlation (*r* = 0.89) between total sugars intake and urinary sucrose and fructose excretion measured from twelve 24 h urine collections per participant. In a study involving twelve subjects living in a metabolic unit and consuming their habitual diets for 30 days Tasevska et al. (15) found that mean daily urinary sucrose and fructose excretion was most strongly associated with intakes of extrinsic sugars (*r* = 0.84) compared to intrinsic sugars (*r* = 0.43). In contrast a recent cohort study of 477 participants in the U.S. self-reported intakes of total sugars were not associated with biomarker-predicted intakes based on single 24-h urine collections (*r* = −0.06) (44). The biomarker predicted intakes were calculated using the formulas developed by Tasevska and colleagues based on feeding studies in a U.K population (13), and were not adapted to a U.S. population. Different sources of sugar, i.e., beet sugars, corn-derived sugars or cane-derived sugars, in the two countries may explain the inconsistent findings.

In our study urinary sugars excretion was assessed using random spot urine collections rather than 24 h urine to reduce respondent burden and we used a sugar specific limited item FFQ that was designed to rank sugar intakes rather to assess actual intakes. More intensive and accurate dietary assessment and 24 h urine collections were not possible in this population study of older Maori adults due to limited funding and our desire to minimize participant burden. It is therefore not surprising that we found weaker correlations between measures than those demonstrated in studies by Tasevska et al. (14, 16) study where multiple days of complete urine samples were collected and analysed. Nevertheless that fact that our analyses showed that are urinary sucrose + fructose biomarker was most strongly associated with sucrose and added sugars, in which glucose and the disaccharides sucrose and fructose would predominate, strengthens our confidence that the biomarker reflects the level of sugars intakes in our population group. The first study reporting on the spot urinary excretion of sucrose and fructose, showed in nine participants in Italy, that the average urinary sucrose excretion of four timed spot urine collections (collected at 8 a.m., 10 a.m., 3 p.m., and 8 p.m.) was correlated with dietary sucrose intake (*r* = 0.70) (45). In a cross-sectional analysis of data from free living participants in the EPIC-Norfolk study (*n* = 475), sucrose intake assessed by FFQ was positively associated with urinary sucrose and fructose excretion in single spot urine samples of individuals with normal body weight (BMI <25 kg/m^2^) (*p* < 0.001). There were, however, no associations shown between urinary sucrose and fructose excretion and self-reported sucrose intake in obese participants (17). Further prospective analysis of prospective data from EPIC Norfolk participants (*n* = 1,734) where sucrose intake was assessed using 7-day diet diaries showed a negative association between sucrose and BMI, whereas sugar intake estimated from baseline spot urine samples was positively associated with BMI. Given that our systematic review of dietary intervention studies showed that sugars intakes are associated with weight gain (8) these findings suggest suggests that urinary excretion of sugars may be a more reliable objective measure of sugars intake than self-report methods in both obese and lean participants. Further previously reported discrepant findings [such as those reported by Bingham et al. (17)] may be due to misreporting of intakes, particularly by obese participants (18). Supporting this theory previous research by Joosen and colleagues, assessing the effect of BMI on urinary sugars excretion, showed that urinary sugars excretion is not affected by BMI (14).

Participants in our study had a high prevalence of overweight and obesity, gout, and dysglycaemia (HbA1C > 40 mmol/mol). Metabolic comorbidities may alter urinary excretion of sugars, affecting the suitability of this biomarker in this group of people. Both diabetes and hyperuricaemia are associated with chronic kidney disease (46, 47) and it is possible that urinary sugars excretion patterns are different for people with these conditions. Furthermore, the mechanism by which sucrose and fructose occurs in the urine is not well-understood (13) except in the case of glucosuria, which is a direct result of elevated blood glucose. Therefore, further research is needed to assess the validity of urinary sugars excretions as a biomarker of sugars intake in participants with metabolic comorbidities such as obesity, diabetes and gout.

Stable isotope ratios in blood have been proposed previously as a biomarker of sugars intake in the U.S. Most of this research evaluated the use of δ^13^C in whole blood or serum (12, 20, 22–28, 30). However, Choy et al. (29) evaluated carbon stable isotope ratios in RBC's alanine as a more precise marker of sugars intake compared to δ^13^C_RBC_ (29). As high intakes of animal proteins also tend to elevate δ^13^C, this approach attempts to account for confounding of the association between sugar and δ^13^C by dietary protein (31, 34, 48). Choy et al. (29) found in a Native Alaskan population (*n* = 68) that RBC's δ^13^C_alanine_ was strongly correlated with self-reported SSBs intake (*r* = 0.70), added sugars intake (*r* = 0.59) and total sugars intake (*r* = 0.57) independent of animal protein intake. Our study showed weaker but statistically significant associations between RBC δ^13^C_alanine_ and added sugars, sucrose and added sugars in sweetened food in multivariate log linear regression models, but not with total sugars, and sugars from SSBs and raw fruit. While the findings are weak this likely reflects the small sample size and the limitations of our dietary assessment method. Further the lack of association with sugars definitions that included sugars from fruit and dairy sources supports the theory that δ^13^C_alanine_ has promise as a marker of sugar-sweetened foods in New Zealand where cane sugar is the predominant sweetener. The lack of association, however, between RBC δ^13^C_alanine_ and the urinary sugars biomarker is not unexpected at the two biomarkers measure sugars intakes over different timeframes; urinary sugars measure recent intake whereas RBC δ^13^C_alanine_ is hypothesized to represent intakes over the previous months.

There are a number of limitations to our study. The primary limitation of this study was its small sample size. Urinary measures were obtained for 153 participants recruited, however we only obtained RBC samples from 36 participants for measurement of δ^13^C_alanine_ and δ^13^C_RBC_ as this was an initial exploratory analysis. The collection of a single spot urine sample rather than a 24 h urine sample is a major limitation as it assumes that sugars excretion is consistent throughout the day. Spot urine samples must thus be corrected for urine concentration. This is typically achieved by correction with urinary creatinine or specific gravity assuming consistent daily excretion values across a population. We corrected for urine dilution with urinary creatinine concentrations however the high level of comorbidity and obesity in our older Maori population means this assumption may not be valid. Even with correction for urine concentration spot samples are not particularly reliable and bias will attenuate the true association between urinary sugars and dietary intakes. On the other hand 24 h urine collections place a high burden on study participants, are frequently incomplete, and are challenging to collect in large population studies. Additionally dietary assessment by self-report is also affected by a high degree of reporting bias further attenuating potential associations between self-reported intakes and the biomarkers of interest. We assessed dietary intakes using a sugar-specific FFQ that had been previously validated for use among Māori in NZ as our reference method (36). FFQs are limited to a finite list of foods and are constrained by the ability of participants to accurately report their food intake retrospectively over a long period of time, and are therefore subject to reporting errors (49). Further research including controlled feeding studies, or free-living participants using dietary measures such as 7-day weighed food records is needed to further study these associations. Because our FFQ instrument was designed specifically to estimate sugars intakes we were not able to assess intakes of other sources of ^13^C-enriched foods such as meat and fish intake that may have confounded the associations between δ^13^C_alanine_ and δ^13^C_RBC_ with sugars intake. Additionally, the FFQ was not designed to distinguish between cane and corn-derived sugars and other sweeteners such as honey, maple syrup and beet sugars that are not enriched in ^13^C. However, consumption of these other sugar sources in New Zealand is relatively low (50) and thus is unlikely to substantially affect the association between stable isotope ratios and dietary sugars intake.

## Conclusions

In conclusion, these results show that both urinary sugars excretion and δ^13^C_alanine_ in red blood cells, but not δ^13^C_RBC_, have potential as objective biomarkers of sugars intake in the New Zealand Māori population, where added sugars in foods and beverages are derived predominantly from sugar cane. However, the weak to moderate associations shown indicate that further research is needed in other populations including groups with and without diabetes and pre-diabetes as these conditions may alter the excretion of sugars in urine and sugars metabolism. Further adequately powered research involving more precise methods of dietary assessment such as a 7-day weighed food record is needed in order to confirm these findings.

## Data Availability Statement

The original contributions presented in the study are included in the article/supplementary material, further inquiries can be directed to the corresponding author/s.

## Ethics Statement

The studies involving human participants were reviewed and approved by University of Otago Human Ethics Committee (13/177). The patients/participants provided their written informed consent to participate in this study.

## Author Contributions

LT, TM, and DK conceived and designed the study. DK, AS, and XT performed the laboratory analyses. RH and AS contributed expertise and support for the isotope analyses. LT analyzed the data. JH supported the study with access to the community, and guardianship and monitoring of the data collection. LT, JM, and TM provided academic supervision. LT, TM, and JH obtained funding for the study and LT had overall responsibility for the research. LT, DK, and RM wrote the paper. All authors contributed to the article and approved the submitted version.

## Conflict of Interest

The authors declare that the research was conducted in the absence of any commercial or financial relationships that could be construed as a potential conflict of interest.
